# Frataxin Deficit Leads to Reduced Dynamics of Growth Cones in Dorsal Root Ganglia Neurons of Friedreich’s Ataxia YG8sR Model: A Multilinear Algebra Approach

**DOI:** 10.3389/fnmol.2022.912780

**Published:** 2022-06-13

**Authors:** Diana C. Muñoz-Lasso, Belén Mollá, Jhon J. Sáenz-Gamboa, Edwin Insuasty, Maria de la Iglesia-Vaya, Mark A. Pook, Federico V. Pallardó, Francesc Palau, Pilar Gonzalez-Cabo

**Affiliations:** ^1^Chemical Biology Group, Department of Biomedical Engineering, Eindhoven University of Technology (TU/e), Eindhoven, Netherlands; ^2^Department of Genetics, Faculty of Biological Sciences, University of Valencia, Valencia, Spain; ^3^CIBER de Enfermedades Raras (CIBERER), Valencia, Spain; ^4^Brain Connectivity Laboratory, Joint Unit FISABIO & Prince Felipe Research Centre (CIPF), Valencia, Spain; ^5^Regional Ministry of Health in Valencia, Hospital Sagunto (CEIB-CSUSP), Valencia, Spain; ^6^CIBER de Salud Mental (CIBERSAM), Valencia, Spain; ^7^The Mathworks B.V., Eindhoven, Netherlands; ^8^Biosciences, Brunel University London, Uxbridge, United Kingdom; ^9^Department of Physiology, Faculty of Medicine and Dentistry, University of Valencia, Valencia, Spain; ^10^Biomedical Research Institute INCLIVA, Valencia, Spain; ^11^Department of Genetic and Molecular Medicine IPER, Institut de Recerca Sant Joan de Déu, Hospital Sant Joan de Déu, Barcelona, Spain; ^12^Division of Pediatrics, University of Barcelona School of Medicine and Health Sciences, Barcelona, Spain

**Keywords:** growth cone, DRG neurons, neurobiology of the disease, Friedreich’s ataxia, tensor decompositions, multilinear algebra

## Abstract

Computational techniques for analyzing biological images offer a great potential to enhance our knowledge of the biological processes underlying disorders of the nervous system. Friedreich’s Ataxia (FRDA) is a rare progressive neurodegenerative inherited disorder caused by the low expression of frataxin, which is a small mitochondrial protein. In FRDA cells, the lack of frataxin promotes primarily mitochondrial dysfunction, an alteration of calcium (Ca^2+^) homeostasis and the destabilization of the actin cytoskeleton in the neurites and growth cones of sensory neurons. In this paper, a computational multilinear algebra approach was used to analyze the dynamics of the growth cone and its function in control and FRDA neurons. Computational approach, which includes principal component analysis and a multilinear algebra method, is used to quantify the dynamics of the growth cone (GC) morphology of sensory neurons from the dorsal root ganglia (DRG) of the YG8sR humanized murine model for FRDA. It was confirmed that the dynamics and patterns of turning were aberrant in the FRDA growth cones. In addition, our data suggest that other cellular processes dependent on functional GCs such as axonal regeneration might also be affected. Semiautomated computational approaches are presented to quantify differences in GC behaviors in neurodegenerative disease. In summary, the deficiency of frataxin has an adverse effect on the formation and, most importantly, the growth cones’ function in adult DRG neurons. As a result, frataxin deficient DRG neurons might lose the intrinsic capability to grow and regenerate axons properly due to the dysfunctional GCs they build.

## Introduction

Computational methods for analyzing biological time-lapse experiments are accelerating the pace of research of neurological diseases every day. In this context, the application of linear algebra methods is well established. Recently, Goodhill et al. (2015) have used principal component analysis (PCA) to analyze the dynamics of growth cone (GC) morphology. In this paper, firstly, we validate the use of PCA to determine principal motion patterns of the GC. Secondly, a generalization of the methods described in Goodhill et al. (2015), is presented by implementing multilinear algebra approaches using tensor decompositions to quantify the dynamics of the growth cone morphology in the neurological disease: Friedreich’s ataxia.

The growth cone (GC) is a sensitive and motile structure accountable for directing and guiding axons and neurites through the tissues and ensuring an accurate connection with their target cells. It appears at the tip of growing axons and neurites during neurite development or axonal regeneration. The GC morphology changes quickly in response to attractive and repulsive clues. It is characterized by presenting two parts, a broad, flattened structure called lamellipodia and thin peaks called filopodia. Moreover, three regions are distinguished based on cytoskeletal content: a central domain (C) rich in microtubules; a peripheral domain (P) enriched with actin filaments; and a transition zone consisting of an area at the interface of the P and C domains. Consequently, when a GC extends, retracts or turns, the cytoskeletal components within the GC are reorganized.

The growth cone moves toward its target in three phases: protrusion, obstruction, and consolidation. First, the lamellipodia and filopodia extend (protrusion). Secondly, the lamellipodia swells while the growth cone swallows it (obstruction). Third, the growth cone narrows and becomes a new axon or neurite (consolidation).

Computational multilinear algebra approaches preserve the spatial-temporal data structures for the analysis, avoiding the vectorization process required by the classical PCA. For this reason, multilinear algebra methods and tensor decompositions for signal and image processing have been applied successfully in medical technology. Yahyanejad et al. (2019) have provided a review of recent applications of tensor analysis methods for biological systems. Also, in bioinformatics, Omberg et al. (2007) used tensor higher-order singular value decomposition for integrative analysis of DNA microarray data. Additionally, in medical imaging, Mathew and Kumar applied Multilinear Principal Component Analysis (MPCA) in order to reduce the dimensionality of the raw data for the classification of diseases using Support Vector Machines (Mathew and Vijaya Kumar, 2019). Furthermore, in neuroscience, Miwakeichi et al. (2004) have applied tensor decompositions to electroencephalographic (EEG) data to identify spatial and temporal patterns.

To study the dynamics of neuronal morphology, researchers typically design time-lapse experiments in order to record axonal growth. Time-lapse experiments record images with two spatial coordinates and time as the third coordinate. In this paper, the use of tensor decompositions is extended to identify the main spatial patterns of the dynamics of growth cone morphology in control and Friedreich’s Ataxia neurons (FRDA, OMIM 229300, ORPHA 95).

The FRDA is a rare progressive neurodegenerative inherited disorder affecting the Caucasian population with two to four individuals per 100,000 (Polo et al., 1991; López-Arlandis et al., 1995). The most common mutation in FRDA patients is a homozygous guanine-adenine-adenine (GAA) trinucleotide repeat expansion in the first intron of *FXN* gene encoding frataxin, which leads to a reduced expression of this protein in all the cells. FRDA is characterized by the dorsal root ganglia (DRG) neurodegeneration through a wholly unknown mechanism. Nevertheless, previous findings, including our research, suggest a link between the DRG neurons’ growth cone (GC) and the pathological hallmarks of FRDA. FRDA cells have impaired mitochondrial ATP production (González-Cabo and Palau, 2013; Bolinches-Amorós et al., 2014), increased oxidative stress (Rodríguez et al., 2020b), calcium dysregulation (Mollá et al., 2017; Rodríguez et al., 2020a) and cytoskeletal destabilization (Pastore et al., 2003; Piermarini et al., 2016; Mollá et al., 2017; Muñoz-Lasso et al., 2020b), which turn on to be essential to regulate the motility of GC and the axonal pathfinding (McCormick and Gupton, 2020; Muñoz-Lasso et al., 2020a).

This paper focuses on studying the dynamics of GC morphology in FRDA. Given the spatial-temporal nature of the GC dynamics, this fundamental question is addressed by applying a well-established computational linear algebra approach (principal component analysis), and extending it by using tensor decompositions to quantify the dynamics of the GC cone morphology. These promise to open a new research direction in imaging and tool development and it is hoped that they will contribute to an enhanced understanding of the role of GC in neurodegenerative diseases.

## Materials and Methods

### Animals

Dr. Mark Pook donated YG8sR and Y47R mice used in this study. The YG8sR and control mice are available in The Jackson Laboratory Repository (YG8sR, Stock No: 024113 and Y47R, Stock No: 024097). The YG8sR mouse model has been derived from natural breeding of YG8R mouse. YG8sR mice are homozygous for a mutation in the *Fxn* gene (knock-out for the *Fxn* allele; *Fxn*^–^) and hemizygous for the YG8s transgene (a copy of the *F.X.N.* gene with ∼250–300 repeats of the GAA triplet). Compared to other human FXN YAC transgenic rescue mouse models such as the YG8R mouse, the YG8sR model expresses contracted human frataxin, resulting in much more significant FXN deficiency (Anjomani Virmouni et al., 2015).

The mouse model was amplified by crossing YG8sR mice with a single copy of the YG8s transgene (*Fxn*^–/–^; *FXN*^±^) with the control Y47R mice containing a single copy of the YG8 transgene containing 9 regular repetitions of the GAA triplet on an *Fxn*^–/–^ background. Animals were maintained and genotyped as previously described (Anjomani Virmouni et al., 2015). For this study, only males were included in the tests. All procedures were carried out following the United Kingdom Home Office’ Animals (Scientific Procedures) Act 1986’ and with approval from the Brunel University Animals Welfare and Ethical Review Board.

### Primary Culture of Adult Dorsal Root Ganglia Neurons

Y47R and YG8sR mice were euthanized by cervical dislocation following the laboratory animals’ care and use guidelines. Whole DRGs were dissected from the entire length of the vertebral column and maintained in ice-cold L-15 (Leibovitz) medium. DRGs were incubated with collagenase and 2.5% Trypsin, then washed in Nutrient Mixture F-12 Ham (Sigma), followed by gentle mechanical trituration in 1 ml of medium. Isolated neurons were pelleted by centrifugation (59 g for 8 min) through a 15% bovine serum albumin (BSA) cushion (BSA 30%: HBSS−/− in a ratio 1:1). Neurons were suspended in media and then seeded either in glass-bottom dishes (MatTek) at 10 neurons/mm^2^, or in microfluidic chambers (Xona Microfluidics, SND150) at a density of 3,5 × 10^4^ neurons per device (see Supplementary Methods for a detailed protocol). Dishes and microfluidic devices were previously coated with 0.5 mg/mL of poly-DL-Ornitin and 0.01 mg/mL of laminin (Sigma). Glass bottom dishes were filled with 2 mL of growth medium and supplemented with mouse nerve growth factor (mNGF, 10 ng/mL, Peprotech bioNova), and mouse glial cell line-derived neurotrophic factor (mGDNF, 10 ng/mL).

### Time-Lapse Experiment

Primary cultures in glass-bottom dishes were incubated during 12–14 h at 37°C, 5% CO_2_. Phase-contrast images of neurons and their GCs were acquired with a LD Achroplan 40×/0.60 dry objective (Zeiss) for 1 h at 30-s intervals with an incubator-inverted microscope (Axiovert 200 M, Zeiss).

### Preparation of the Non-plasma Bonded Devices

We prepared the microfluidic devices following the instructions recommended by Xona Microfluidics, Inc. and choosing the best conditions for primary culture of dorsal root ganglia neurons. The first step is sterilizing the Corning (24 mm × 40 mm) cover glasses. For this, we placed them in a stainless-steel staining rack and immersed them in a glass staining dish full of 95% ethanol (sealed with parafilm). Later, we sonicated them in a water bath sonicator for 30 min. We left the slides to dry in a biosafety cabinet overnight. The next day, we coated the cover glasses with 0.5 mg/mL Poli-DL-Ornitin (PDL, Sigma-Aldrich). To do this, we put the sterile cover glasses in sterile 100 mm dishes (2 per each 10 cm petri dish) and added 1 mL of the sterile 0.5 mg/ml PDL solution on each cover glass and incubated overnight. The next day, we proceeded to rinse the glass slides three times with sterile dH_2_O (taking care they do not overlap), placed each cover glass in sterile 60 mm dishes, and filled the dish with 3 mL sterile dH_2_O (cover slices are covered) and incubated for 3 h. Immediately after, we rinsed the cover glass with sterile dH_2_O three more times, then aspirated off the water and allowed the glass slides to air-dry overnight in a bio-safety cabinet. We assembled the coated cover glasses for the Silicone Device the same day. For this, we first removed any debris remaining on the Silicone Device by using 3M™ Scotch Brand 471 Vinyl tape. Then, we proceeded to sterilize the Silicone Devices by rinsing them with 70% ethanol and allowing them to air dry the feature side up for 1 h in the bio-safety cabinet, ensuring that the Devices were thoroughly dry before proceeding. To assemble the devices, we put each PDL coated glass into a new sterile 60 mm dish and placed each device evenly on top of each coated glass using two tweezers (previously sterilized by autoclave). After that, we added 150 μl of laminin to the top left well, allowed the laminin to enter the main channel for 1 min, and then filled the top right well with 150 μl of laminin. Returned to the incubator and incubated overnight. The next day, the day of primary culture, we removed the laminin. We added 150 μl to the top right well, allowed the media to enter the main channel for 1 min, then filled the bottom right well with 150 μl of media and returned to the incubator 2–3 h (the time expended to isolate the DRG neurons).

### *In vitro* Axotomy

Primary culture of DRGs isolated from mice with 2 months of age in microfluidic chambers was performed and maintained at 37°C in a humidified 5% CO_2_ incubator as described above. The medium was supplemented with murine NGF and GDNF as follows; Days 0–1: 100 ng/ml of each neurotrophin; Days 1–3: somal compartment, 50 ng/ml; axonal compartment, 100 ng/ml; Day 3 onwards: somal compartment, 25 ng/ml; axonal compartment, 100 ng/ml and were grown for 48 h. Axotomy was performed at 3 DIV (days *in vitro*) by transecting the axons away from the cell bodies with suction for 5 s with a fine tip glass pipette. The axonal compartment was immediately washed and replaced with a fresh medium. Then, cultures were incubated for 2 days.

Images of SOMA and AXON chambers were acquired at different steps of the *in vitro* axotomy. Five DIV (before axotomy), immediately after, and 48 h after axotomy. Phase-contrast images of cell bodies (SOMA chamber) and axonal network (AXON chamber) were acquired at 40× with an incubator-inverted microscope (Axiovert 200 M, Zeiss).

### Morphometric Analysis of the Growth Cone

We established and followed customized parameters to quantify the growth cone’s morphology (GC) (Goodhill et al., 2015) based on our expertise and others. We chose to analyze the morphology of the growth cone with phase-contrast imaging with *in vivo* conditions to diminish the effect of external factors other than the reduced expression of frataxin in DRG neurons. Images were captured of entire neurons to choose the GC at the tip of the thickest neurite, which probably corresponds to the axon. We selected GCs that were not overlapping with other neurites/axons or debris. Furthermore, we chose to analyze GC exhibiting a hand-like shape.

Images were processed and analyzed with the Fiji/Image-J software (N.I.H). A polygon selection was manually traced following the peripheral region of each GC, and the measurement tool was executed. We selected what we considered the 4 most biological relevant parameters: the area, Feret’s diameter, circularity and solidity. On the one hand, the area and Feret’s diameter (the longest distance between any two points along the selection boundary) reflect the GC’s size. The circularity and solidity are shape descriptors that reflect the geometry of the GC. Circularity (4π*area/perimeter2) indicate how circular is the GC, a value of 1.0 indicates a perfect circular GC. As values approach 0, it means a more elongated GC. Besides, solidity is the ratio of GC area over their convex area; then, it shows how much a GC fills the area of its filopodial spread (Chitsaz et al., 2015).

### Analysis of Axonal Growth and Regeneration in Microfluidic Chambers

Phase-contrast images were acquired with *in vivo* conditions and processed following the method proposed by Sasaki et al. (2009) with some modifications. Images were acquired from random fields in the axon chamber from three independent experiments. Phase-Contrast images were processed with the Analyze particles – plugin of Fiji/ImageJ (N.I.H) software (Schindelin et al., 2012) to obtain the measurements of the total and the fragmented area of the axonal network. Later, we calculated the degeneration index as the ratio of the fragmented area and total area as proposed by Sasaki et al. (2009). In order to use the Analyze particle plugin, we had to establish some conditions and parameters based on those settled by Sasaki et al. (2009), (Supplementary Figure 4). First, we extracted the minimum 5 and maximum of 10 quadrangular regions (700 pixels × 700 pixels) from images of separated fields of the axonal chamber. Therefore, per each genotype, a minimum of 15 and a maximum of 30 quadrangular regions were analyzed (*N* = 3 mice per genotype, Supplementary Table 1). Those areas where the axons were stacking were excluded for this analysis. In order to optimize the image processing and avoid biases, images were ordered in stacks based on similarities of the grayscale and then processed with the Process module of Fiji/ImageJ (NIH) to increase contrast and resolution with the steps described next: (a) Filters: Median (radius: 0.2 pixels), (b) Subtract background (Rolling ball radius: 5 pixels, Light background, Sliding paraboloid), (c) Enhance contrast (Saturated pixels: 0.3%, Normalize, Equalize histogram). Later we obtained binary images following the next steps and parameters: (a) Threshold (Method: Otsu), Black and White (B&W), Light background (dark background inactive).

Once we got the binary images, we proceeded to obtain the measurements for the axonal network’s fragmented and total area. To calculate the fragmented area, we used the analyze particle plugin of Fiji/ImageJ (N.I.H) following the next steps and parameters: (a) particle size: 20–10000 Pixel Units, (b) circularity: 0.5–1.0, (c) exclude analysis at edges: yes. To quantify the total area, we obtained the total sum of pixels (RawIntDen) for each binary-region with the R.O.I. manager tools of Fiji/ImageJ (NIH). We calculated the degeneration index with these data, which consisted of dividing the area occupied by the axonal network’s fragments by the value of the total area occupied by the axonal network. The parameters settled here were slightly adjusted from those proposed by Sasaki et al. (2009), who used the primary culture of DRGs isolated from embryonic mice as a model.

### Motion Tracking Analysis With MtrackJ

The GCs were imaged every 30 s for 1 h using Zeiss Axiovision software. Active GCs (spread growth cones, not retracted) and growing in all directions were chosen for these measurements. Therefore, we handle all sequence images to get a common reference point. Sequence images of GCs were extracted from the original files. Then, they were rotated to get the base of the growth cone centered on the *x*-axis and the GC (filopodia and lamellipodia) pointing to the right. The base of the GCs was tracked manually using the MtrackJ plugin (Meijering et al., 2012). A 5 min time interval was chosen to avoid variability (Goodhill et al., 2015). The average value obtained for length, velocity and α [°] in each track (one-time lapse per GC) was used for statistical analysis. α [deg] is the angle of the in-plane component of the most recent displacement vector (pointing from the previous point to the current point of the track) with respect to the *x*–*y*- coordinate system of the image (with the origin taken in the previous point). The values range from −180 to +180 degrees, where 0 degrees means the vector component runs parallel to the positive *x*-axis (pointing to the right), +90 degrees (or −90 degrees) means it runs parallel to the positive (or negative) *y*-axis (pointing downward, or upward, respectively), and +180 degrees (which is the same as −180 degrees) means it runs parallel to the negative *x*-axis (pointing to the left).

### Study of the Growth Cone Dynamics by Principal Component Analysis

The dynamics of the growth cone morphology is complex and difficult to quantify due to the size, the shape and/or the position of the growth cone change within minutes (Goldberg and Burmeister, 1986; Goodhill et al., 2015). Here, we have implemented the method based on Principal Component Analysis (PCA) proposed by Goodhill et al. (2015) with customized modifications.

The methodology included several steps (Figure 3A). First, we obtained segmented images from the time-lapse experiments. To do this, we developed a customized platform in MATLAB^®^ (The MathWorks, Inc.) that allowed us to process the experiments semi-automatically (∼1 h per time-lapse) (Supplementary Figure 3). We captured the growth cone outline from the segmented time-lapse experiments and obtained a list of coordinates (*x*-, *y*-). At this point, we got coordinates that represented each growth cone outline as a single point in a 500-dimensional space. Last, we performed a PCA algorithm in MATLAB^®^ to obtain the directions of the space’s main patterns. The two main components explaining most of the data’s variance were visualized using the bi-plot algorithm in MATLAB^®^.

### Tensor Decomposition Analysis

The method proposed by Goodhill et al. (2015) is based on the application of principal component analysis (PCA), which is fundamentally suitable for 2-dimensional data. The image sequence of living cells can be stacked as spatial temporal 3-dimensional data structures, and 4–dimensional data structures if we consider an additional coordinate for the type of cells. In linear algebra, tensors are the generalizations of vectors and matrices for multiple coordinates. For this reason, tensors are the most convenient mathematical form to handle the 3-dimensional or 4-dimensional data structures generated by time-lapse biology experiments. Traditionally in PCA (a technique for 2-dimensional data), the images (which are spatial data structures in two coordinates) are converted into vectors (1-dimensional data structures) and then stacked in a data matrix (2-dimensional data structure). This process destroys the spatial structure and the spatial correlations relevant to the subsequent analysis. This section has applied methods based on multilinear algebra and tensor analysis to overcome this limitation. Tensors are multidimensional generalizations of the concept of vectors and matrices. Since the 2-dimensional spatial structure in the images is preserved, it is also possible to maintain the correlation space-time in a sequence of images.

Similarly to the matrix’s case, there is a multilinear principal component analysis (MPCA) used to find principal patterns of movement in image sequences. Particularly in cell biology, there are no references to applying these numerical techniques to analyze growth cones. The algorithms (see Supplementary Methods) have been programed in MATLAB^®^ based on (de Lathauwer et al., 2000).

## Results

### Growth Cones From Frataxin Deficient Dorsal Root Ganglia Neurons Show Abnormal Morphology

**FIGURE 1:**
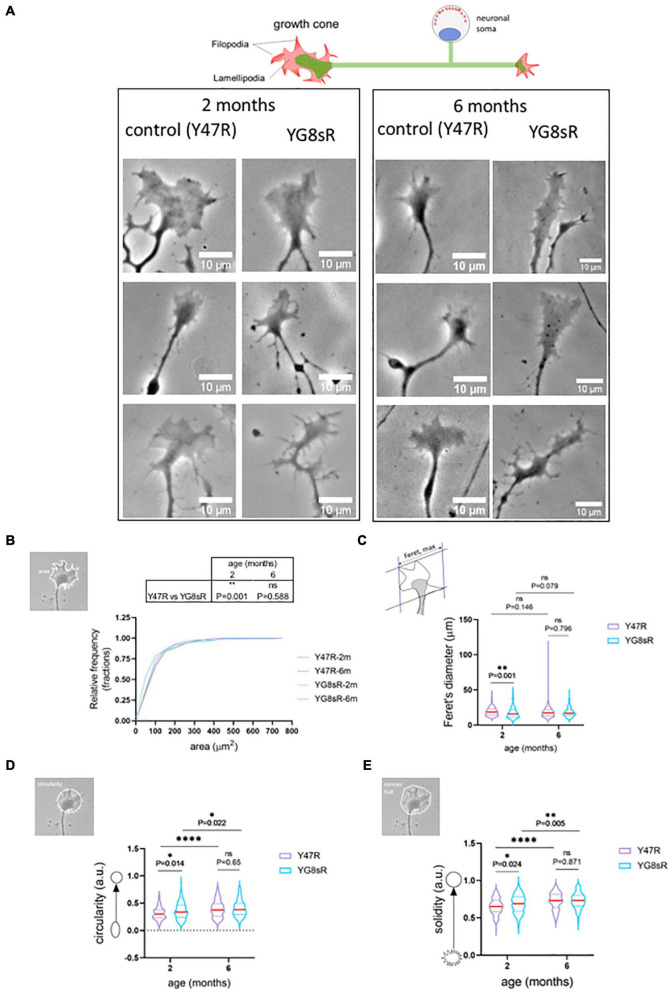
Growth cones of frataxin deficient neurons of the YG8sR mouse are smaller and abnormal. **(A)** Representative phase-contrast images of three SPREAD like GCs observed in the primary culture of DRG neurons from the control (Y47R) and the YG8sR model at 2 and 6 months of age. We observed disorganization in the distribution of filopodia and lamellipodia of YG8sR-GCs. Notice the hand-like shape in the controls and the absence of this shape in the YG8sR-GCs. Also, we observed small structures, probably rich in actin, growing from the axon or neurite close to the YG8sR-GCs (black arrows). These structures are not observed in the controls. Images were acquired with *in vivo* conditions after 14–16 h of culture. Each image is representative of independent experiments (three mice per each genotype and age). Scale bar, 10 μm. **(B)** Cumulative distribution of the growth cone area was plotted for both genotypes at 2 and 6 months of age. Most of the growth cones of DRG neurons from the YG8sR mice were smaller (0–100 μm^2^) than those from the controls at 2 months of age (Y47R: 150 growth cones from *n* = 3, YG8sR: 133 growth cones from *n* = 3, ^**^P = 0.001, Mann–Whitney test). This difference is lost at 6 months of age (Y47R: 147 growth cones from *n* = 5, YG8sR: 154 growth cones from *n* = 4, *P* = 0.001, Mann–Whitney test, *P* = 0.587). **(C–E)** Shape parameters of the GCs. **(C)** The GCs from the YG8sR mice at 2 months of age were smaller **(C)** (Y47R: 150 growth cones from *n* = 3, YG8sR: 133 growth cones from *n* = 3, ^**^P = 0.001), more circular **(D)** (Y47R: 150 growth cones from *n* = 3, YG8sR: 133 growth cones from *n* = 3, *P = 0.014) and more convex **(E)** (Y47R: 150 growth cones from *n* = 3, YG8sR: 133 growth cones from *n* = 3, *P = 0.024). These differences are lost at 6 months of age (Y47R: 147 growth cones from n = 5, YG8sR: 154 growth cones from *n* = 4, *P* = 0.001, Mann–Whitney test, *P* = 0.587). The data distribution is presented with violin plots. The median (red horizontal bar) and interquartile (25 and 75%, colored horizontal bars) are shown. Figures beside the *y*-axis of the graphs visualize examples of the shape descriptors calculated. Statistical significance: ^****^P-value < 0.0001.

The murine models that emulate the mutation responsible for the FRDA present a series of complications, mainly because the genomic environment is different. Overall, they show signs of the start of the neurodegeneration cascade; however, the dorsal root ganglia (DRG), dentate nucleus (DN), or cerebellum do not degenerate as observed in the pathology of the human disease, probably because of the short lifespan of murine models. On the other hand, these models, particularly the YG8R/YG8sR mice (Mollá et al., 2017; Muñoz-Lasso et al., 2020a), have been broadly characterized by several researchers who have determined that they are suitable for investigating the first neuropathological changes occurring in the FRDA and establishing the molecular and cellular mechanisms that underlie the neurodegenerative process. We previously showed that GCs of frataxin-deficient neurons from a 24 months old mice model for FRDA, the YG8R mouse, were smaller and showed qualitative morphological changes compared to their controls (Muñoz-Lasso et al., 2020a). Consequently, we aimed to confirm quantitatively whether these morphological observations were present in the living GCs of adult neurons isolated from YG8sR mice and determine their evolution. To quantify changes in the GCs of DRG neurons, we performed phase-contrast imaging with *in vivo* conditions of the dorsal root ganglia’s primary culture (DRGs) (Supplementary Figure 1). This approach allowed us to analyze the morphology of the GCs from DRG neurons at 2 and 6 months of age in a natural state without affecting neurons’ viability (Figure 1A). As described in methods, we manually selected the GCs following the GC-outline in Fiji/ImageJ (NIH) of the images acquired and got measurements for several shape parameters: circularity, Feret’s diameter, solidity, and area.

At 2 months of age we found that 73.3% of the mice YG8sR-GCs had an area between 0 and 100 μm^2^, contrasting with the 78.9% obtained for the control GCs (Figure 1B). Then, mice YG8sR-GCs showed a reduced area compared with control neurons GCs (YG8sR: 105.74 ± 15.83 μm^2^; control: 110.16 ± 9.66 μm^2^; ^**^P-value = 0.001) and reduced Feret’s diameter (YG8sR: 17.56 ± 0.71 μm^2^; control: 19.27 ± 0.51 μm; *P*-value = 0.001) (Figure 1C). Furthermore, the mice YG8sR-GCs were more circular (YG8sR: 0.36 ± 0.02; control: 0.31 ± 0.02; *P*-value = 0.014) (Figure 1D) and were characterized for showing a solid geometry (YG8sR: 0.69 ± 0.02; control: 0.65 ± 0.02; *P*-value = 0.024) (Figure 1E) compared to the controls.

Significant differences were not detected at 6 months for any shape parameters, including the area and Feret’s diameter (Table 1 and Figures 1B–D). These results are explained by the high variability of the data observed at 6 months of age in both genotypes (Table 1), particularly in the control mice. Furthermore, the control and YG8sR data distributions are similar (Figures 1B–D) for any shape parameters tested.

**TABLE 1:** Overview of the data obtained for the morphometric analysis of the growth cone.

2 months						

	Control (Y47R)			YG8sR		
	Mean	SEM	*N*	Mean	SEM	*N*
	126.44	11.30	50	108.16	17.47	48
Area (μm^2^)	99.30	7.55	50	116.78	19.09	33
	104.75	10.15	50	92.29	10.92	52
**Total mean**	110.16	9.66	*N* = 150	105.74	15.83	*N* = 133
	20.10	0.94		17.03	1.01	
Feret’s diameter (μm)	18.13	0.80		19.54	1.82	
	19.58	0.92		16.79	1.04	
**Total mean**	19.27	0.89		17.78	1.29	
	0.32	0.01		0.38	0.02	
Circularity (a.u.)	0.32	0.02		0.34	0.02	
	0.30	0.02		0.36	0.02	
**Total mean**	0.31	0.02		0.36	0.02	
	0.67	0.02		0.71	0.02	
Solidity (a.u)	0.66	0.02		0.67	0.02	
	0.63	0.02		0.68	0.02	
**Total mean**	0.65	0.02		0.69	0.02	

**6 months**						

	**Control (Y47R)**			**YG8sR**		
	**Mean**	**SEM**	***N***	**Mean**	**SEM**	***N***

	127.43	115.59	12	84.64	48.39	33
	121.65	72.11	21	134.53	112.55	51
Area (μm^2^)	110.43	59.57	35	130.11	74.12	46
	157.84	124.70	22	99.63	61.37	24
	145.93	278.25	57			
**Total mean**	132.66	130.04	*N* = 147	112.23	74.11	*N* = 154
	20.07	1.77		15.04	0.76	
	17.67	1.15		19.11	1.00	
Feret’s diameter (μm)	18.46	0.91		20.01	1.07	
	20.81	1.79		17.92	1.34	
	18.69	1.93				
**Total mean**	19.14	1.51		18.02	1.04	
	0.29	0.03		0.42	0.03	
Circularity (a.u.)	0.39	0.03		0.39	0.02	
	0.34	0.02		0.41	0.02	
	0.38	0.03		0.40	0.02	
	0.45	0.02				
**Total mean**	0.37	0.03		0.40	0.02	

These results confirm that frataxin deficiency produces morphological alterations in the GCs of DRG neurons of early young adult mice, at 2 months of age, and generates aberrant morphology.

### Frataxin Deficiency Reduces the Neurite Extension Rate and Alters the Growth Cone Turning in Adult Dorsal Root Ganglia Neurons

**FIGURE 2:**
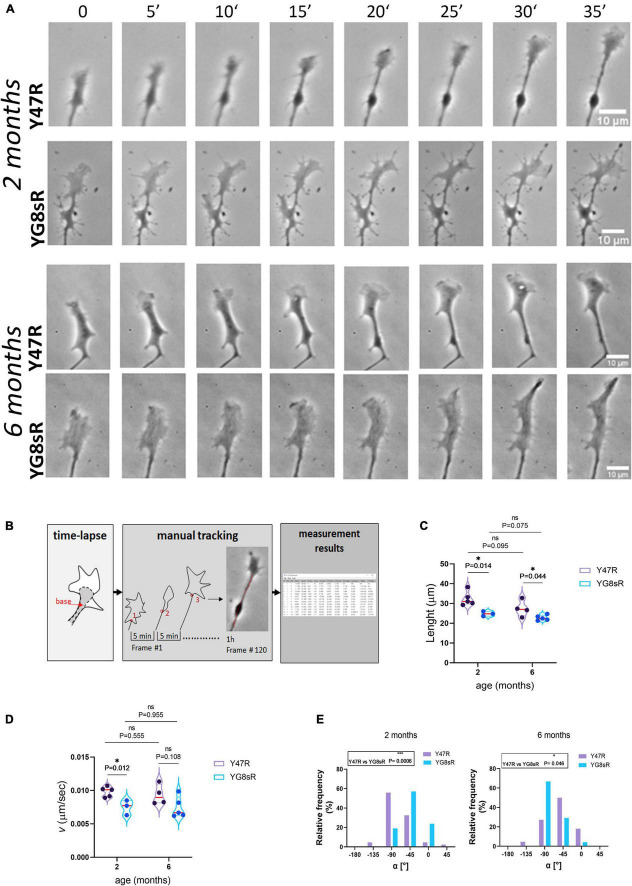
Neurites of frataxin-deficient neurons of the YG8sR mouse grow slower and with not defined trajectories. **(A)** Representative sequences of frames 5 min apart from a time-lapse movie of control and YG8sR- growth cone (GC) at 2 and 6 months of age. Scale bar, 10 μm. **(B)** Workflow to obtain the motion tracking with the MTrackJ plugin. First, rotation of the movie to a fixed position to start. Secondly, selection of the points of the track with the points located at the center of the base of the growth cone (red points) and in frames 5 min apart; and Third, obtention of the final measurement results. **(C–E)** Violin plots show the average Length **(C)** and velocity **(D)** data’s general distribution. **(E)** Graphical representation of the frequency distributions for the GC’s average turning angles (α[°]) at 2 (left, Y47R: 42 growth cones from *n* = 3, YG8sR: 21 growth cones from *n* = 3) and 6 months of age (right, Y47R: 19 growth cones from *n* = 3, YG8sR: 24 growth cones from *n* = 3). The GCs of YG8sR neurons travel fewer distances with lower speed and distinctive turning angles than the controls at 2 (^***^: exact *P*-value = 0.0006) and 6 (*: exact *P*-value = 0.046). The median (red horizontal bar) and interquartile (25 and 75%, colored horizontal bars) are shown. Statistical significance was determined using the Holm–Sidak method for length and velocity and using the Mann–Whitney test for the turning angle.

The GCs of DRG neurons are highly motile structures that change their morphology quickly during growth (Supplementary Video 1). Because the morphology and the function of the growth cone are directly connected, we were aimed to determine whether the aberrant morphology observed for the adult YG8sR-GCs was also affecting the GC-dynamics by measuring the speed, displacement and GC-turning events. To reach this goal, here we used Time-Lapse phase-contrast microscopy to record living GCs (Figure 2A) and a particle tracking plugin: *MTrackJ* (Meijering et al., 2012) for tracking the GC base (Figure 2B and see Section “Materials and Methods” for detailed explanation).

We choose to track the base of the GC (Figure 2B) because, (i) it is easy to detect and, therefore, (ii) the position of the base changes as soon as the GC advances (Supplementary Videos 1–6). Before performing the manual tracking, all time-lapse experiments were orientated so that the GC’s base was centered to the *x*-axis and pointing to the right, so there is a common point of reference for all the time-lapse experiments (see Section “Materials and Methods”).

We found that YG8sR-GCs traveled reduced distances at 2 (control: 32.39 ± 4.13 μm; YG8sR: 24.81 ± 3.14 μm; *P*-value = 0.014) and 6 months (control: 27.45 ± 4.31 μm; YG8sR: 22.75 ± 2.89 μm; *P*-value = 0.044) of age compared to the controls (Table 2 and Figure 2C). Furthermore, we observed a significant decrease in the velocity of displacement at 2 months of age (control: 0.010 ± 0.001 μm/sec; YG8sR: 0.008 ± 0.001 μm/sec; *P*-value = 0.012) that was lost at 6 months of age (control: 0.009 ± 0.002; YG8sR: 0.007 ± 0.001; *P*-value = 0.108) (Figure 2D).

**TABLE 2:** Overview of the data obtained from the quantification of the growth cone dynamics.

2 months						

	Control (Y47R)			YG8sR		
	Mean	SEM	*N*	Mean	SEM	*N*
Velocity (μm/sec)	0.010	0.002	9	0.009	0.001	4
	0.009	0.001	7	0.008	0.001	7
	0.011	0.002	10	0.006	0.001	10
	0.010	0.001	5			
	0.009	0.001	11			
**Total mean**	0.010	0.001	*N* = 42	0.008	0.001	*N* = 21
Length (μm)	29.26	4.76		23.61	3.57	
	31.04	3.17		26.05	2.93	
	38.31	5.61		24.76	2.92	
	33.17	4.20				
	30.18	2.94				
**Total mean**	32.39	4.13	*N* = 42	24.81	3.14	*N* = 21
Turning angle (°)	−63.15	10.95		−38.34	21.46	
	−66.09	9.73		−44.53	12.95	
	−82.89	7.24		−43.73	5.81	
	−63.20	25.55				
	−62.43	6.65				
**Total mean**	−67.55	12.02	*N* = 42	−42.20	13.41	*N* = 21

**6 months**						

	**Control (Y47R)**			**YG8sR**		
	**Mean**	**SEM**	***N***	**Mean**	**SEM**	***N***

Velocity (μm/sec)	0.011	0.003	3	0.010	0.002	7
	0.010	0.003	3	0.006	0.001	5
	0.008	0.002	4	0.008	0.002	6
	0.008	0.001	9	0.006	0.001	3
				0.007	0.001	3
**Total mean**	0.009	0.002	*N* = 19	0.007	0.001	*N* = 24
Length (μm)	32.82	7.57		23.39	1.06	
	22.92	1.48		21.81	3.18	
	26.66	5.95		24.72	5.40	
	27.39	2.24		22.50	1.73	
				21.31	3.06	
**Total mean**	27.45	4.31	*N* = 19	22.75	2.89	*N* = 24
Turning angle (°)	−45.22	23.55		−66.30	6.76	
	−71.43	24.74		−81.08	2.96	
	−56.58	6.96		−78.36	6.86	
	−59.48	11.02		−73.81	19.10	
				−41.19	28.17	
**Total mean**	−58.18	16.56	*N* = 19	−68.15	12.77	*N* = 24

Since GC turning is also a critical behavior that allows the GCs to seek the correct synaptic target, we were aimed to analyze GC turning angles in our time-lapse experiments. By analyzing the average angle of displacement of each GC during the time-lapse, we observed that the GC turning angles of the YG8sR-GCs differ significantly from neurons from the control mice at 2 (*P*-value = 0.0006) and 6 (*P*-value = 0.046) months of age. At 2 months, while 24% of the YG8sR-GCs turned horizontally (0°, −180°, +180°) and 57% diagonally (−45°, +45°), 57% of the controls turned vertically (−90°, +90°) and 31% diagonally (Figure 2E, left and Table 2). At 6 months, while 67% of the YG8sR-GCs turned vertically and 29% diagonally, 32% of the controls turned vertically and 47% diagonally (Figure 2E, right and Table 2). Then, at 2 months, YG8sR-GCs turning is limited to horizontal and diagonal turnings compared to the control. A broader distribution of turning angles is observed (Supplementary Videos 1–6).

Interestingly, the YG8sR-GCs turning patterns are lost at 6 months. However, it remains distinct from the turning patterns observed for the controls. Overall, the changes in the spatial behavior of YG8sR-GCs suggest that frataxin deficient YG8sR neurons can build GCs, but these would be dysfunctional.

### Frataxin Deficiency Reduces the Dynamics of the Growth Cone Morphology of Adult Dorsal Root Ganglia Neurons

Previously, we have shown a morphological alteration of the GCs of frataxin deficient neurons from the YG8sR mouse model. These observations are significant at 2 months of age but it is lost at 6 months of age. Interestingly, we have also shown that YG8sR-GC has reduced motility, which is more prominent at 2 months by tracking the GC’base (Figure 2B), they did not include the GC’s macrostructural features (e.g., filopodia and lamellipodia). Therefore, we chose to quantify the neuronal GC morphology dynamics with two computer-based methods in MATLAB^®^ (The Mathworks). The significant advantages of using these methods are the capacity to analyze extensive datasets and the obtention of important information of the GC morphology lost with the previous manual tracking of the GC’s base. Therefore, it is essential to mention that with these computer-based methods, we can get valuable information on changes in the whole GCs, including the filopodia. The reason is that here we are detecting changes in the time of the entire structure of the GC.

We investigated the behavior of GCs at 2 months of age by applying the methods to the same time-lapse experiments used for the manual approach described above. The first method is a Principal Components Analysis (PCA) (Goodhill et al., 2015), which uses matrix analysis fundamentally suitable for 2-dimensional data, with customized modifications (see Section “Materials and Methods” and Supplementary Data 1 for details of the algorithms). To perform PCA analysis of the images, sequences of living cells, which are spatial-temporal data structures in 2 coordinates, were converted into vectors (1-dimensional data structures) and then stacked in a data matrix (2-dimensional data structure).

**FIGURE 3:**
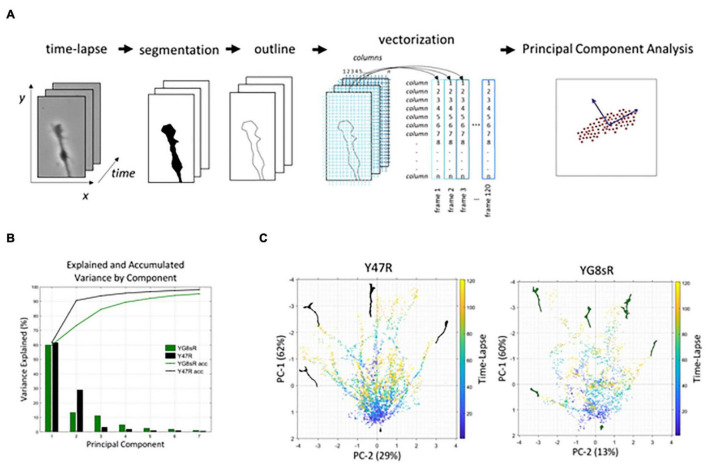
The PCA analysis shows that YG8sR growth cones have a reduced dynamic morphology at 2 months of age. **(A)** Workflow used for the principal component analysis. **(B)** The principal component analysis showed that components 1 and 2 explain 91 and 73% of the variance for the control and YG8sR GCs, respectively. **(C)** Bi-plots of components 1 and 2 visualizing the movement of the growth cone for 1 h. The principal components indicate the main patterns of movement of all points that form the growth cone, including the axonal segment that the growth cone builds over the time-lapse. The lines start in the lower central part of the images (initial position of the cones at *t* = 0) and are directed to the right, left or above in the images with different angles (final position of the cones at *t* = 60 min). Therefore, the more dispersed the points are in the graphs, there are fewer trajectories of movement. The length of each trajectory indicates the distance traveled by the growth cones in the time-lapse. When compared to the controls, the YG8sR-growth cones exhibited fewer trajectories and traveled short distances. Besides, the trajectories of the YG8sR-growth cones are less defined and more dispersed.

Figure 3A shows the steps that were followed in the process. First, we obtained binary images from the phase-contrast time-lapse experiments (segmentation). In doing so, we created a semi-automated platform to get binary images for each time-lapse with MATLAB^®^ (The MathWorks) to optimize the image’s processing and reduce the variability introduced by the observer (see Section “Materials and Methods” for more details). Secondly, we obtained an outline of the GC that was later processed to get each GC’s vectorization in the time-lapse (Figure 3A). Finally, the PCA is performed with these vectorized data. PCA-analysis results showed that components 1 and 2 explain 91 and 73% of the variance for the control and YG8sR GCs, respectively (Figure 3B). The 2D scatter plots of these two components (Figure 3C) show that control GCs have long trajectories (concentration of dots giving shape to lines in the graph) of movement in several directions (from −180° to 180°). These trajectories are characterized as a well-defined path from the beginning (Figure 3C, left graph), possibly indicating the high exploratory and growth activity of the control GCs. On the contrary, the YG8sR-GCs show mostly short trajectories with random and curved movement patterns (Figure 3C, right graph), with absent well-defined paths indicating weak exploratory activity and low growth capacity.

**FIGURE 4:**
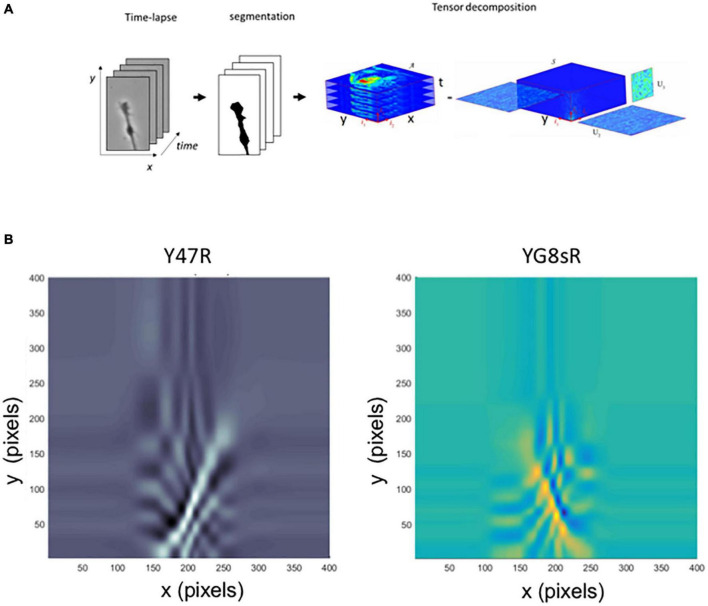
A tensor analysis confirms the reduced dynamics of DRG neurons’ growth cone morphology from the YG8sR mouse. **(A)** Visualization of the methodology applied for the tensor analysis. **(B)** Graph of the two principal components obtained for tensor analysis. Like the PCA analysis, the principal components indicate the main patterns of movement of all points that form the growth cone, including the axonal segment built during the time-lapse. These patterns are seen in the graphs as continuous lines that stand out from the background color. The lines start in the lower central part of the images (initial position of the cones at *t* = 0) and are directed to the right, left or above in the images with different angles (final position of the cones at *t* = 60 min). When the main components of the YG8sR mouse (right) and the control are compared (left), we observed that movement patterns of the growth cones of the YG8sR mouse (yellow and blue) are different from those of the control, presenting shorter movement patterns directed to the right or left (horizontal).

From a mathematical perspective, classical PCA methods are based on matrix analysis and are not best suited to identify patterns in spatial-temporal phenomena such as time-lapse microscopy. To overcome this limitation, we have used unconventional numerical methods based on Tensor analysis. Tensors are multidimensional generalizations of the concept of vectors and matrices. Since the 2-dimensional spatial structure in the images is preserved, it is also possible to safeguard the correlations space-time in a sequence of images (Figure 4A).

Similarly to the matrix’s case, a multilinear principal component analysis (MPCA) is used to find principal movement patterns in image sequences. There are no references to applying these numerical techniques to analyze growth cones, particularly cell biology. The code to compute the tensor decompositions we have programmed in MATLAB^®^ is based on the Tensor Toolbox work (de Lathauwer et al., 2000; Brett and Tamara, 2021) (Supplementary Data 2, 3). The results show that the pattern of movement of the control-GCs was mostly vertical (Figure 4B, left). In contrast, the YG8sR-GCs, showed combined vertical and horizontal lines, indicating a differential and insufficient exploratory activity of the GCs (Figure 4B, right).

The results obtained with the PCA and tensor analysis coincide in showing alterations of the dynamics of the GCs of frataxin deficient neurons at 2 months of age. These observations may explain the reduced dynamics observed when we manually tracked the GC base.

### Adult Frataxin Deficient Dorsal Root Ganglia Neurons Lose Their Intrinsic Ability to Regenerate *in vitro*

As a consequence of the morphological and functional changes observed in the growth cones (GCs) of YG8sR-DRG neurons, it was possible to postulate that critical cellular processes, such as axonal regeneration, were also being affected. In axonal regeneration, peripheral neurons can reactivate axonal growth programs, including axonal elongation and axonal pathfinding, active during the development. Besides, neurons rely on axonal transport to clear the debris or recycle damaged proteins and ensure the new axon’s materials. Therefore, the growth cone (GC) plays a vital role in the unique capability of peripheral axons to regenerate and reinnervate their target tissues and organs after an injury during adulthood (Blanquie and Bradke, 2018; Muñoz-Lasso et al., 2020b).

**FIGURE 5:**
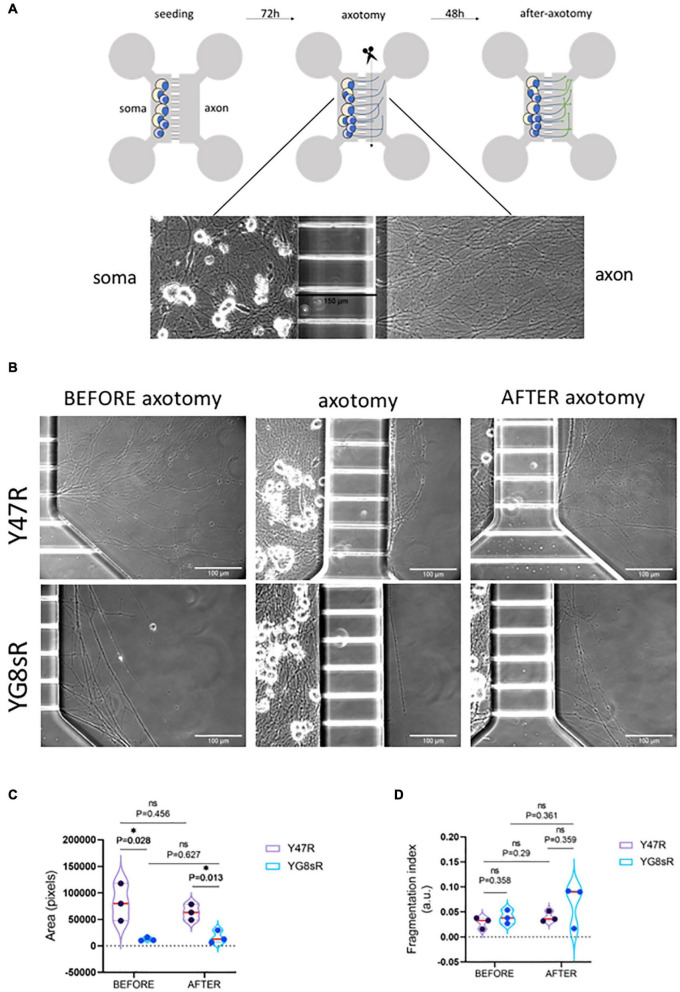
Adult DRG neurons from the YG8sR mouse reduce their natural regenerative response after an injury *in vitro.* **(A)** Scheme of a microfluidic device (upper panel) and steps of the experiment (bottom panel). Each device has two identical compartments connected with microfluidic channels (150 μm in length). Neuronal somas were seeded into one of the chambers (soma-chamber), and were allowed to grow under a neurotrophic gradient pushing them to grow their axons crossing the microchannels. Axotomy (or cutting of axons) was performed 3 days in culture, and neurons were cultured for 2 days more. Images show a typical view of the neuronal somas and axons in the device after 3 days of culture. **(B)** Representative images of control and YG8sR-living axons during the experiment. Before (3 days in culture), immediately after axotomy (3 days in culture), and after axotomy (2 days after axotomy). Compared to the controls (top-left), few DRG neurons of the YG8sR-mice were able to cross the microchannels and extend axonal networks (bottom-left). After the axotomy, DRG neurons of the YG8sR-mice tried to regenerate the axons with visible difficulty. **(C)** Violin plots show the distribution of the means obtained for the area covered by the axons before and after the axotomy. Data show a reduced area covered by the axons of DRG neurons of the YG8sR-mice before and after the axotomy. The area covered by the control axons is visibly higher before and after the axotomy. **(D)** Violin plots show the distribution of the means obtained for the fragmentation index (fragmented area versus total area) before and after the axotomy. Data show a non-significant tendency to increase the fragmented area in the control and YG8sR axons. All the means corresponded to three independent experiments and were obtained by averaging the values collected from 10 random fields in each experiment’s axonal chamber. The median (red horizontal bar) and interquartile (25 and 75%, colored horizontal bars) are shown. Significance was determined using an unpaired *t*-test with the Holm–Sidak method. Statistical significance: *P-value < 0.05.

To investigate whether the DRG-neurons from YG8sR mouse model can extend healthy axons and regenerate appropriately after an injury, we performed an axotomy *in vitro* as a model for axonal damage (Figure 5A). Here, we have chosen to evaluate DRG neurons’ regenerative response at 2 months of age because we have shown throughout this work that GCs of YG8sR-DRG are significantly defective at this age. Also, a high regenerative capacity is expected for young neurons. An equivalent number of neuronal somas isolated from the YG8sR and control mice were plated in the soma chambers (3 × 10^4^ neurons per device) for the experiment. Then, neurons were progressively exposed to a neurotrophic gradient for 3 days (see Section “Materials and Methods” for details). Later, we performed the axotomy and then allowed the axons to regenerate for 2 days. Phase-Contrast Microscopy with *in vivo* conditions was used to capture and examine the morphology of living neurons and their axons.

After 3 days in culture and before performing the axotomy, we observed a considerably reduced number of axons in YG8sR-DRG neurons than in the control neurons (Figure 5B). In addition to this, we observed that axons of the YG8sR-DRG neurons that crossed the microchannels were stacked once they passed the microchannel contrasting with the axonal arrays observed in the control neurons (Figure 5B and Tables 3, 4).

**TABLE 3:** Data obtained for the quantification of the degeneration index of the control mice.

Control (Y47R) 2 months pre-axotomy (*N* = 3)						

# fields analyzed	Fragmented area (μm^2^)		Total axon area		Degeneration index (DI)	
	Mean	SEM	Mean	SEM	Mean	SEM
10	1704,7	106,5	117819,6	12341,0	0,016	0,002
10	2540,0	170,5	79655	6168,3	0,033	0,003
10	1685,6	129,3	47331,8	5766,4	0,038	0,003

**Control (Y47R) 2 months post-axotomy (*N* = 3)**						

**# fields analyzed**	**Fragmented area (μm^2^)**		**Total axon area**		**Degeneration index (DI)**	
	**Mean**	**SEM**	**Mean**	**SEM**	**Mean**	**SEM**

10	2766,7	108,7	78336,6	5215,4	0,016	0,002
10	2461,5	109,8	48792,2	3937,5	0,033	0,003
10	1976,8	134,8	63100,4	4315,5	0,038	0,002

**TABLE 4:** Data obtained for the quantification of the degeneration index of the YG8sR mice.

Control (YG8sR) 2 months pre-axotomy (*N* = 3)						

# fields analyzed	Fragmented area (μm^2^)		Total axon area		Degeneration index (DI)	
	Mean	SEM	Mean	SEM	Mean	SEM
7	538.2	78.0	10676.4	1693.1	0.054	0.007
6	372.2	114.7	10185.2	1999.9	0.038	0.007
10	419.7	42.4	16390.0	2319.8	0.027	0.003

**Control (YG8sR) 2 months post-axotomy (*N* = 3)**						

**# fields analyzed**	**Fragmented area (μm^2^)**		**Total axon area**		**Degeneration index (DI)**	
	**Mean**	**SEM**	**Mean**	**SEM**	**Mean**	**SEM**

7	1128.1	100.3	12660.1	1359.3	0.092	0.008
6	397.7	116.5	6384.3	1631.4	0.090	0.034
9	453.4	56.8	29539.1	4357.5	0.017	0.002

The objective was to determine how the low density of axons could affect the regenerative capacity of YG8sR-DRG neurons and find out whether the axotomy stimulates other axons to cross the microchannels. After 2 days of performing the axotomy, we observed that YG8sR-DRG neurons could not regenerate their axons as the control axons did it. Also, the axotomy did not produce additional axons to cross the microchannels. Next, we evaluate signs of degeneration such as axonal swelling and axonal fragmentation in the axons that crossed the microchannels. To do this, we performed a quantification method proposed by Sasaki et al. (2009) based on Phase-Contrast Microscopy and DRG culture from mouse embryos to measure an axonal degeneration index. This method uses the Particle analyzer algorithm of Fiji/ImageJ for detecting particles with shape and size characteristic of degenerated axons (see Section “Materials and Methods”). Our data confirmed our previous observations, showing that YG8sR-DRG neurons respond poorly to the neurotrophic gradient and grow less axons than control neurons (Figure 5C). However, we did not detect significant differences in the degeneration index before or after the axotomy (Figure 5D).

Besides the axons, we were also interested in evaluating the neuronal somas after the axotomy. Interestingly, we did not observe noticeable changes in the number or the morphology of the cell bodies that evidenced a process of cell death (Supplementary Figure 2). Overall, our results show that while control DRG neurons can grow healthy axons with well-defined tracks in response to a neurotrophic gradient, the YG8sR-DRG neurons cannot do it. This behavior is maintained even after the axotomy and without affecting neuronal cell bodies, which do not show changes in the number or the morphology of the cell bodies that evidenced a process of cell death (Supplementary Figure 2). All of them suggest that adult YG8sR-DRG neurons have reduced regenerative potential compared to control neurons, and the molecular mechanism involved probably is axonal-specific.

## Discussion

In this work, a link between the growth cone, a critical neuronal structure, and the pathophysiology of FRDA is revealed by using *in vivo* phase-contrast imaging and computational methods in the YG8sR mouse. When an axon grows or regenerates, the mitochondrial network, the neuronal cytoskeleton and motor proteins are carefully coordinated to create a growth cone (GC). The GC plays a critical role in guiding axons to their targets. They navigate to their targets, driven by several molecular clues that trigger the reorganization of the GC-cytoskeleton producing changes in the morphology and determining which way the growth cone will turn and direct the new axon’s growth (Muñoz-Lasso et al., 2020b).

The model of degeneration described in the FRDA involves a distal degeneration of peripheral sensory neurons, with initial deterioration of synapses, slowly progressing to the spinal cord’s posterior columns and corticospinal and spinocerebellar tracts. This model of degeneration of the dying-back type is the most common form of axonal degeneration described in the PNS. Nevertheless, there is increasing evidence that the neurodegeneration process has to be very early in life (Indelicato et al., 2018) or even during neurodevelopment (Koeppen et al., 2017). Nerve fiber loss in patients is heterogeneous and correlates with the size of the short GAA allele. However, there is no association between the severity of the neuropathy and the duration of the disease. This suggests that the loss of neurons is early and stable over time. Indeed, a neuropathological study of tissue samples from FRDA patients supports the conclusion that it is during development that the dorsal root is altered, due to inappropriate growth and guidance of the axons of neural crest-derived neurons (Koeppen et al., 2017). We think our findings support this hypothesis. We observed that frataxin deficient DRG neurons from the YG8sR mice built smaller and morphologically aberrant GCs than their controls at 2 months. These significant morphological changes were not observed in mice at 6 months of age. Therefore, morphological alterations on the GCs might appear at embryonic stages when long axons of DRG neurons are actively being produced. Although it is speculative, our results suggest that developmental defects may induce the neurodegenerative process that determines the observations made by these authors. This hypothesis has to be confirmed at an early postnatal age.

The morphology and function of the GCs are tightly connected. Consequently, the YG8sR-GCs function was observed to be reduced and aberrant. These results indicate that the GCs of frataxin deficient DRG neurons indeed have reduced motility compared to the control GCs at 2 and 6 months of age. This reduced motility is characterized by a reduced speed of migration, broader turning angles and more short and complex trajectories, indicating that frataxin-deficient DRG neurons can create GCs, but they are dysfunctional.

The knowledge about the FRDA’s neurons’ GC dynamics is lacking, showing evidence of a functional affection of the DRG-GCs in an FRDA mouse model. Despite the fact that several quantitative approaches to measure the dynamics of the morphology of the GCs are available, most of them imply technical limitations. First, high-resolution microscopy allows quantifying individual filopodia present in the GCs; nevertheless, it is a difficult task and time-consuming. It relies heavily on human intuition to determine the most significant variance in the growth cone morphology (Goodhill et al., 2015). Secondly, the use of fluorescent probes facilitates the tracking and increases the resolution of the filopodia; then, images from fixed cells or *in vivo* experiments can be analyzed using computer-based tools to quantify the filopodia automatically. However, fixation can alter filopodia’s real amount and the architecture of the GCs. In addition, adult DRG neurons are not easy to transfect or transduce, even at young ages, such as 2 months of age. Several transfection methods of adult DRG neurons produce high cell death levels (glial cells and neurons) proportional to the mice’s age. Thus, the use of fluorescent proteins or probes could induce changes in the morphology of the growth cone that would have no relation with frataxin producing misleading results. In contrast, Phase-Contrast microscopy with *in vivo* conditions reduces external factors to the media, neurotrophic elements, and frataxin deficit.

YG8sR-GCs grow smaller neurites and exhibit altered dynamics compared to control-GCs by using two computer-based methods, PCA and Tensor Decomposition analysis. Despite the fact that additional efforts are required to reveal the molecular changes associated with these findings, these two-practical computer-based approaches can quickly be adapted to further analyses of the GC motility in the FRDA and other disorders of the nervous system, which is an unexplored field due to the GC spatial-temporal complexity.

Among the potential implications of dysfunctional GCs in the frataxin-deficient DRG neurons was the affection of axonal growth programs such as axonal pathfinding and axonal regeneration, which ensure the proper development and maintenance of DRG neurons throughout their lifetime. These two critical processes were investigated using an *in vitro* axotomy of DRG axons as a model for damage. After dissection of DRGs, the intuitive ability to grow axons *in vitro* was not affected in both the control and YG8sR-DRG neurons. However, after the axotomy, the YG8sR-neurons did not regenerate their axons to the extent the controls did. Additionally, damage or evident loss of neuronal cell bodies of YG8sR neurons was not apparent, before and after the axotomy; these results indicate that an axonal specific disturbing condition might prevent the proper formation of functional GCs. In conclusion, frataxin deficient DRG neurons from the YG8sR mice may lose the intrinsic ability to regenerate their axons after injury, a protective phenomenon observed mainly in DRG neurons (McQuarrie and Grafstein, 1973; Richardson and Issa, 1984). A possible explanation for this observation is the failure of axonal regeneration programs in the YG8sR-DRG neurons, which in normal conditions allows them to protect against cumulative axon damage.

The low capacity of YG8sR axons to cross the microfluidic channels and their inability to explore the axon area is striking. Within the microfluidic chamber, axons are incited to cross the microchannels by physical (volume of the media), and, more importantly, a neurotrophic gradient of GDNF and NGF. Previous works have shown the therapeutic potential of different neurotrophic factors for Friedreich’s ataxia, including insulin-like growth factor-1 (IGF-1) (Franco et al., 2012; Sanz-Gallego et al., 2014), Brain-derived Neurotrophic Factor (BDNF) (Katsu-Jiménez et al., 2016) and the range of neuroprotective factors secreted by mesenchymal stem cells (MSCs) (Kemp K. C. et al., 2017; Kemp K. et al., 2017). Therefore, we have to consider that the presence of neurotrophins in the experiments of this work may be masking a more pronounced alteration than that observed. GDNF and NGF are neurotrophic factors well known to stimulate axonal regeneration and pathfinding in peripheral axons *in vitro* and *in vivo* (Menesini Chen et al., 1978; Fine et al., 2002; Dudanova et al., 2010; Schuster et al., 2010; Mashanov et al., 2020). Furthermore, GDNF and NGF have been shown to modulate other pathfinding pathways such as sema3A pathway in adult DRG neurons (Wanigasekara and Keast, 2006). Therefore, we cannot exclude that the inability of the YG8sR-DRG to cross the microchannels may reflect a defect on both neurotrophin and axonal pathfinding pathways such as sema3A.

In the FRDA pathophysiology context, the affection of axonal pathfinding pathways and axonal regeneration programs in DRG neurons implies that several axons could not reach their target tissues effectively. Consequently, the innervation of DRG neurons would be inefficient. The evaluation of nerve biopsies from FRDA patients (McLeod, 1971; Santoro et al., 1999; Nolano et al., 2001; Indelicato et al., 2018) supports this hypothesis. In addition to this, the evidence of pathological axonal phenotypes observed in necropsies studies on FRDA patients proposed that FRDA neuropathology could be a developmental delay (Koeppen et al., 2017). How and why this problem occurs during development remains to be solved. Our results support the hypothesis that an incorrect innervation could occur due to axonal growth programs alteration, such as axonal pathfinding pathways and axonal regeneration. When an axon cannot innervate correctly, it can be eliminated through physiological neuronal death (Clarke and Cowan, 1976). Therefore, both a dysfunctional GC and the inability to regenerate axons against cumulative damage explain why the DRG is a subject of a hypoplastic process.

On the other hand, grumose degeneration with abnormal proliferation of its synaptic terminals involving Purkinje cells has been described in the cerebellum of patients (Koeppen et al., 2015; Kemp et al., 2016). It has recently been suggested that grumose degeneration could be a survival mechanism for Purkinje cells to generate new axons and establish contact with other surviving neurons (Koeppen et al., 2015). Again, if the axonal regeneration process is altered in these neurons, the possibility of generating new contacts with the efferent targets would be reduced, and therefore retrograde atrophy would be facilitated.

Further analysis of the GC dynamics and axonal regeneration response is needed to reveal the cascade of molecular events that leads to the dysfunction of the DRG-GC in the FRDA. In this context, the morphology and motility of GC rely on three critical central functions of the mitochondria that are known to be affected in FRDA cells: (a) ATP production, (b) the maintenance of cellular redox status and (c) buffering of cytosolic calcium [Ca^2+^]_*i*_. Interestingly, evidence from patients and cellular and animal models show that FRDA cells present alterations in these three processes. Defects on mitochondrial dynamics (Puccio et al., 2001; Hick et al., 2013; Bolinches-Amorós et al., 2014) and neuronal cytoskeleton (Pastore et al., 2003; Sparaco et al., 2009; Piermarini et al., 2016; Muñoz-Lasso et al., 2020a) have been described. Moreover, recent studies have settled the Ca^2+^-mediated signaling and homeostasis in the sequence of pathologic events observed in FRDA neurons. Defects in the Ca^2+^ buffering capacity have been observed in neurons (Bolinches-Amorós et al., 2014; Mincheva-Tasheva et al., 2014; Mollá et al., 2017; Purroy et al., 2018) and cerebellar granule neurons from the YG8R mice (Abeti et al., 2018).

In summary, we have evidenced an adverse effect of the deficit of frataxin in the formation and, most importantly, the growth cones’ function in adult DRG neurons by applying both classical and novel computer-assisted analysis. As a result, we propose that frataxin deficient DRG neurons might lose the intrinsic capacity to grow and regenerate axons properly due to the dysfunctional GCs they build.

## Data Availability Statement

The datasets presented in this study can be found in online repositories. The names of the repository/repositories and accession number(s) can be found in the article/Supplementary Material.

## Ethics Statement

The animal study was reviewed and approved by Brunel University Animals Welfare and Ethical Review Board.

## Author Contributions

DM-L conducted and designed experiments, analyzed the results, and wrote the manuscript. BM performed the experiments. MP provided the mouse model and funding acquisition. EI performed the Tensor Analysis in MATLAB^®^ (Mathworks) and wrote the manuscript. MI-V and JS-G performed the PCA analysis. FVP interpreted the data and wrote the manuscript. FP funding acquisition, interpreted the data, and wrote the manuscript. PG-C designed the study, supervised the experiments, analyzed the data, funded acquisition, and wrote the manuscript. All authors read and approved the final manuscript.

## Conflict of Interest

EI was employed by company The Mathworks B.V. The remaining authors declare that the research was conducted in the absence of any commercial or financial relationships that could be construed as a potential conflict of interest.

## Publisher’s Note

All claims expressed in this article are solely those of the authors and do not necessarily represent those of their affiliated organizations, or those of the publisher, the editors and the reviewers. Any product that may be evaluated in this article, or claim that may be made by its manufacturer, is not guaranteed or endorsed by the publisher.

## Supplementary Material

Supplementary Video 1 Phase-contrast imaging of living growth cone two months old isolated from the first Y47R mouse.

Supplementary Video 2 Phase-contrast imaging of living growth cone two months old isolated from the second Y47R mouse.

Supplementary Video 3 Phase-contrast imaging of living growth cone two months old isolated from the third Y47R mouse.

Supplementary Video 4 Phase-contrast imaging of living growth cone two months old isolated from the first YG8sR mouse.

Supplementary Video 5 Phase-contrast imaging of living growth cone two months old isolated from the second YG8sR mouse.

Supplementary Video 6 Phase-contrast imaging of living growth cone two months old isolated from the third YG8sR mouse.

## References

[B1] Abeti R. Brown A. F. Maiolino M. Patel S. Giunti P. (2018). Calcium deregulation: novel insights to understand Friedreich’s ataxia pathophysiology. *Front. Cell. Neurosci.* 12:264. 10.3389/fncel.2018.00264 30333728 PMC6176067

[B2] Anjomani Virmouni S. Ezzatizadeh V. Sandi C. Sandi M. Al-Mahdawi S. Chutake Y. (2015). A novel GAA-repeat-expansion-based mouse model of Friedreich’s ataxia. *Dis. Model. Mech.* 8 225–235. 10.1242/dmm.018952 25681319 PMC4348561

[B3] Blanquie O. Bradke F. (2018). Cytoskeleton dynamics in axon regeneration. *Curr. Opin. Neurobiol.* 51 60–69. 10.1016/j.conb.2018.02.024 29544200

[B4] Bolinches-Amorós A. Mollá B. Pla-Martín D. Palau F. González-Cabo P. (2014). Mitochondrial dysfunction induced by frataxin deficiency is associated with cellular senescence and abnormal calcium metabolism. *Front. Cell. Neurosci.* 8:124. 10.3389/fncel.2014.00124 24860428 PMC4026758

[B5] Brett W. B. Tamara G. K. (2021). *Tensor Toolbox for MATLAB^®^, Version 3.2.1.* Available online at: www.tensortoolbox.org

[B6] Chitsaz D. Morales D. Law C. Kania A. (2015). An automated strategy for unbiased morphometric analyses and classifications of growth cones *in vitro*. *PLoS One* 10:e0140959. 10.1371/journal.pone.0140959 26496644 PMC4619750

[B7] Clarke P. G. Cowan W. M. (1976). The development of the isthmo-optic tract in the chick, with special reference to the occurrence and correction of developmental errors in the location and connections of isthmo-optic neurons. *J. Comp. Neurol.* 167 143–164. 10.1002/cne.901670203 58875

[B8] de Lathauwer L. de Moor B. Vandewalle J. (2000). A multilinear singular value decomposition. *SIAM J. Matrix Anal. Appl.* 21 1253–1278. 10.1137/S0895479896305696

[B9] Dudanova I. Gatto G. Klein R. (2010). GDNF acts as a chemoattractant to support ephrinA-induced repulsion of limb motor axons. *Curr. Biol.* 20 2150–2156. 10.1016/j.cub.2010.11.021 21109439

[B10] Fine E. G. Decosterd I. Papaloïzos M. Zurn A. D. Aebischer P. (2002). GDNF and NGF released by synthetic guidance channels support sciatic nerve regeneration across a long gap. *Eur J. Neurosci.* 15 589–601. 10.1046/j.1460-9568.2002.01892.x 11886440

[B11] Franco C. Fernández S. Torres-Alemán I. (2012). Frataxin deficiency unveils cell-context dependent actions of insulin-like growth factor i on neurons. *Mol. Neurodegener.* 7:51. 10.1186/1750-1326-7-51 23039828 PMC3547778

[B12] Goldberg D. J. Burmeister D. W. (1986). Stages in axon formation: observations of growth of Aplysia axons in culture using video-enhanced contrast-differential interference contrast microscopy. *J. Cell Biol.* 103 1921–1931. 10.1083/jcb.103.5.1921 3782290 PMC2114395

[B13] González-Cabo P. Palau F. (2013). Mitochondrial pathophysiology in Friedreich’s ataxia. *J. Neurochem.* 126(Suppl. 1), 53–64. 10.1111/jnc.12303 23859341

[B14] Goodhill G. J. Faville R. A. Sutherland D. J. Bicknell B. A. Thompson A. W. Pujic Z. (2015). The dynamics of growth cone morphology. *BMC Biol.* 13:10. 10.1186/s12915-015-0115-7 25729914 PMC4353455

[B15] Hick A. Wattenhofer-Donzé M. Chintawar S. Tropel P. Simard J. P. Vaucamps N. (2013). Neurons and cardiomyocytes derived from induced pluripotent stem cells as a model for mitochondrial defects in Friedreich’s ataxia. *Dis. Model. Mech.* 6 608–621. 10.1242/dmm.010900 23136396 PMC3634645

[B16] Indelicato E. Nachbauer W. Eigentler A. Rudzki D. Wanschitz J. Boesch S. (2018). Intraepidermal nerve fiber density in Friedreich’s ataxia. *J. Neuropathol. Exp. Neurol.* 77 1137–1143. 10.1093/jnen/nly100 30358880

[B17] Katsu-Jiménez Y. Loría F. Corona J. C. Díaz-Nido J. (2016). Gene transfer of Brain-derived Neurotrophic Factor (BDNF) prevents neurodegeneration triggered by FXN deficiency. *Mol. Ther.* 24 877–889. 10.1038/mt.2016.32 26849417 PMC4881769

[B18] Kemp K. Dey R. Cook A. Scolding N. Wilkins A. (2017). Mesenchymal stem cell-derived factors restore function to human frataxin-deficient cells. *Cerebellum* 16 840–851. 10.1007/s12311-017-0860-y 28456899 PMC5498643

[B19] Kemp K. C. Cerminara N. Hares K. Redondo J. Cook A. J. Haynes H. R. (2017). Cytokine therapy-mediated neuroprotection in a Friedreich’s ataxia mouse model. *Ann. Neurol.* 81 212–226. 10.1002/ana.24846 28009062 PMC5324580

[B20] Kemp K. C. Cook A. J. Redondo J. Kurian K. M. Scolding N. J. Wilkins A. (2016). Purkinje cell injury, structural plasticity and fusion in patients with friedreich’s ataxia. *Acta Neuropathol. Commun.* 4:53. 10.1186/s40478-016-0326-3 27215193 PMC4877974

[B21] Koeppen A. H. Becker A. B. Qian J. Gelman B. B. Mazurkiewicz J. E. (2017). Friedreich ataxia: developmental failure of the dorsal root entry zone. *J. Neuropathol. Exp. Neurol.* 76 969–977. 10.1093/jnen/nlx087 29044418 PMC6440497

[B22] Koeppen A. H. Ramirez R. L. Becker A. B. Feustel P. J. Mazurkiewicz J. E. (2015). Friedreich ataxia: failure of GABA-ergic and glycinergic synaptic transmission in the dentate nucleus. *J. Neuropathol. Exp. Neurol.* 74 166–176. 10.1097/NEN.0000000000000160 25575136 PMC4294979

[B23] López-Arlandis J. M. Vílchez J. J. Palau F. Sevilla T. (1995). Friedreich’s ataxia: an epidemiological study in Valencia, Spain, based on consanguinity analysis. *Neuroepidemiology* 14 14–19. 10.1159/000109774 7898602

[B24] Mashanov V. Alwan A. Kim M. W. Lai D. Poerio A. Ju Y. M. (2020). Synergistic effect of CNTF and GDNF on directed neurite growth in chick embryo dorsal root ganglia. *PLoS One* 15:e0240235. 10.1371/journal.pone.0240235 33017447 PMC7535060

[B25] Mathew J. Vijaya Kumar R. (2019). Multilinear principal component analysis with SVM for disease diagnosis on big data. *IETE J. Res.* 68 526–540. 10.1080/03772063.2019.1615008

[B26] McCormick L. E. Gupton S. L. (2020). Mechanistic advances in axon pathfinding. *Curr. Opin. Cell Biol.* 63 11–19. 10.1016/j.ceb.2019.12.003 31927278 PMC7247931

[B27] McLeod J. G. (1971). An electrophysiological and pathological study of peripheral nerves in Friedreich’s ataxia. *J. Neurol. Sci.* 12 333–349. 10.1016/0022-510x(71)90067-0 5550263

[B28] McQuarrie I. G. Grafstein B. (1973). Axon outgrowth enhanced by a previous nerve injury. *Arch. Neurol.* 29 53–55. 10.1001/archneur.1973.00490250071008 4711805

[B29] Meijering E. Dzyubachyk O. Smal I. (2012). Methods for cell and particle tracking. *Methods Enzymol.* 504 183–200. 10.1016/B978-0-12-391857-4.00009-4 22264535

[B30] Menesini Chen M. G. Chen J. S. Levi-Montalcini R. (1978). Sympathetic nerve fibers ingrowth in the central nervous system of neonatal rodent upon intracerebral NGF injections. *Arch. Ital. Biol.* 116 53–84. 655758

[B31] Mincheva-Tasheva S. Obis E. Tamarit J. Ros J. (2014). Apoptotic cell death and altered calcium homeostasis caused by frataxin depletion in dorsal root ganglia neurons can be prevented by BH4 domain of Bcl-xL protein. *Hum. Mol. Genet.* 23 1829–1841. 10.1093/hmg/ddt576 24242291

[B32] Miwakeichi F. Martínez-Montes E. Valdés-Sosa P. A. Nishiyama N. Mizuhara H. Yamaguchi Y. (2004). Decomposing EEG data into space-time-frequency components using Parallel Factor Analysis. *Neuroimage* 22 1035–1045. 10.1016/j.neuroimage.2004.03.039 15219576

[B33] Mollá B. Muñ;oz-Lasso D. C. Riveiro F. Bolinches-Amorós A. Pallardó F. V. Fernandez-Vilata A. (2017). Reversible axonal dystrophy by calcium modulation in frataxin-deficient sensory neurons of YG8R mice. *Front. Mol. Neurosci.* 10:264. 10.3389/fnmol.2017.00264 28912677 PMC5583981

[B34] Muñoz-Lasso D. C. Romá-Mateo C. Pallardó F. V. Gonzalez-Cabo P. (2020b). Much more than a scaffold: cytoskeletal proteins in neurological disorders. *Cells* 9:358. 10.3390/cells9020358 32033020 PMC7072452

[B35] Muñoz-Lasso D. C. Mollá B. Calap-Quintana P. García-Giménez J. L. Pallardo F. V. Palau F. (2020a). Cofilin dysregulation alters actin turnover in frataxin-deficient neurons. *Sci. Rep.* 10:5207. 10.1038/s41598-020-62050-7 32251310 PMC7090085

[B36] Nolano M. Provitera V. Crisci C. Saltalamacchia A. M. Wendelschafer-Crabb G. Kennedy W. R. (2001). Small fibers involvement in Friedreich’s ataxia. *Ann. Neurol.* 50 17–25. 10.1002/ana.1283 11456305

[B37] Omberg L. Golub G. H. Alter O. (2007). A tensor higher-order singular value decomposition for integrative analysis of DNA microarray data from different studies. *Proc. Natl. Acad. Sci. U.S.A.* 104 18371–18376. 10.1073/pnas.0709146104 18003902 PMC2147680

[B38] Pastore A. Tozzi G. Gaeta L. M. Bertini E. Serafini V. di Cesare S. (2003). Actin glutathionylation increases in fibroblasts of patients with Friedreich’s ataxia: a potential role in the pathogenesis of the disease. *J. Biol. Chem.* 278 42588–42595. 10.1074/jbc.M301872200 12915401

[B39] Piermarini E. Cartelli D. Pastore A. Tozzi G. Compagnucci C. Giorda E. (2016). Frataxin silencing alters microtubule stability in motor neurons: implications for Friedreich’s ataxia. *Hum. Mol. Genet.* 25 4288–4301. 10.1093/hmg/ddw260 27516386

[B40] Polo J. M. Calleja J. Combarros O. Berciano J. (1991). Hereditary ataxias and paraplegias in Cantabria, Spain. An epidemiological and clinical study. *Brain* 114(Pt. 2), 855–866. 10.1093/brain/114.2.855 2043954

[B41] Puccio H. Simon D. Cossée M. Criqui-Filipe P. Tiziano F. Melki J. (2001). Mouse models for Friedreich ataxia exhibit cardiomyopathy, sensory nerve defect and Fe-S enzyme deficiency followed by intramitochondrial iron deposits. *Nat. Genet.* 27 181–186. 10.1038/84818 11175786

[B42] Purroy R. Britti E. Delaspre F. Tamarit J. Ros J. (2018). Mitochondrial pore opening and loss of Ca2+ exchanger NCLX levels occur after frataxin depletion. *Biochim. Biophys. Acta Mol. Basis Dis.* 1864 618–631. 10.1016/j.bbadis.2017.12.005 29223733

[B43] Richardson P. M. Issa V. M. (1984). Peripheral injury enhances central regeneration of primary sensory neurones. *Nature* 309 791–793. 10.1038/309791a0 6204205

[B44] Rodríguez L. R. Lapeñ;a T. Calap-Quintana P. Moltó M. D. Gonzalez-Cabo P. Langa J. A. N. (2020b). Antioxidant therapies and oxidative stress in friedreich’s ataxia: The right path or just a diversion? *Antioxidants* 9:664. 10.3390/antiox9080664 32722309 PMC7465446

[B45] Rodríguez L. R. Calap-Quintana P. Lapeñ;a-Luzón T. Pallardó F. V. Schneuwly S. Navarro J. A. (2020a). Oxidative stress modulates rearrangement of endoplasmic reticulum-mitochondria contacts and calcium dysregulation in a Friedreich’s ataxia model. *Redox Biol.* 37:101762. 10.1016/j.redox.2020.101762 33128998 PMC7585950

[B46] Santoro L. de Michele G. Perretti A. Crisci C. Cocozza S. Cavalcanti F. (1999). Relation between trinucleotide GAA repeat length and sensory neuropathy in Friedreich’s ataxia. *J. Neurol. Neurosurg. Psychiatry* 66 93–96. 10.1136/jnnp.66.1.93 9886462 PMC1736186

[B47] Sanz-Gallego I. Torres-Aleman I. Arpa J. (2014). IGF-1 in Friedreich’s Ataxia - proof-of-concept trial. *Cerebellum Ataxias* 1:10. 10.1186/2053-8871-1-10 26331034 PMC4552279

[B48] Sasaki Y. Vohra B. P. S. Lund F. E. Milbrandt J. (2009). Nicotinamide mononucleotide adenylyl transferase-mediated axonal protection requires enzymatic activity but not increased levels of neuronal nicotinamide adenine dinucleotide. *J. Neurosci.* 29 5525–5535. 10.1523/JNEUROSCI.5469-08.2009 19403820 PMC3162248

[B49] Schindelin J. Arganda-Carreras I. Frise E. Kaynig V. Longair M. Pietzsch T. (2012). Fiji: an open-source platform for biological-image analysis. *Nat. Methods* 9 676–682. 10.1038/nmeth.2019 22743772 PMC3855844

[B50] Schuster K. Dambly-Chaudière C. Ghysen A. (2010). Glial cell line-derived neurotrophic factor defines the path of developing and regenerating axons in the lateral line system of zebrafish. *Proc. Natl. Acad. Sci. U.S.A.* 107 19531–19536. 10.1073/pnas.1002171107 20974953 PMC2984216

[B51] Sparaco M. Gaeta L. M. Santorelli F. M. Passarelli C. Tozzi G. Bertini E. (2009). Friedreich’s ataxia: oxidative stress and cytoskeletal abnormalities. *J. Neurol. Sci.* 287 111–118. 10.1016/j.jns.2009.08.052 19748629

[B52] Wanigasekara Y. Keast J. R. (2006). Nerve growth factor, glial cell line-derived neurotrophic factor and neurturin prevent semaphorin 3A-mediated growth cone collapse in adult sensory neurons. *Neuroscience* 142 369–379. 10.1016/j.neuroscience.2006.06.031 16876331

[B53] Yahyanejad F. Albert R. DasGupta B. (2019). A survey of some tensor analysis techniques for biological systems. *Quant. Biol.* 7 266–277. 10.1007/s40484-019-0186-5

