# Supplementary material for: Frataxin Deficit Leads to Reduced Dynamics of Growth Cones in Dorsal Root Ganglia Neurons of Friedreich’s Ataxia YG8sR Model: A Multilinear Algebra Approach
Source: Front Mol Neurosci. 2022 Jun 13;15:912780. doi: 10.3389/fnmol.2022.912780 (PMC9236133; doi:10.3389/fnmol.2022.912780)
Supplement: Supplementary file 7 [file Image_1.pdf]

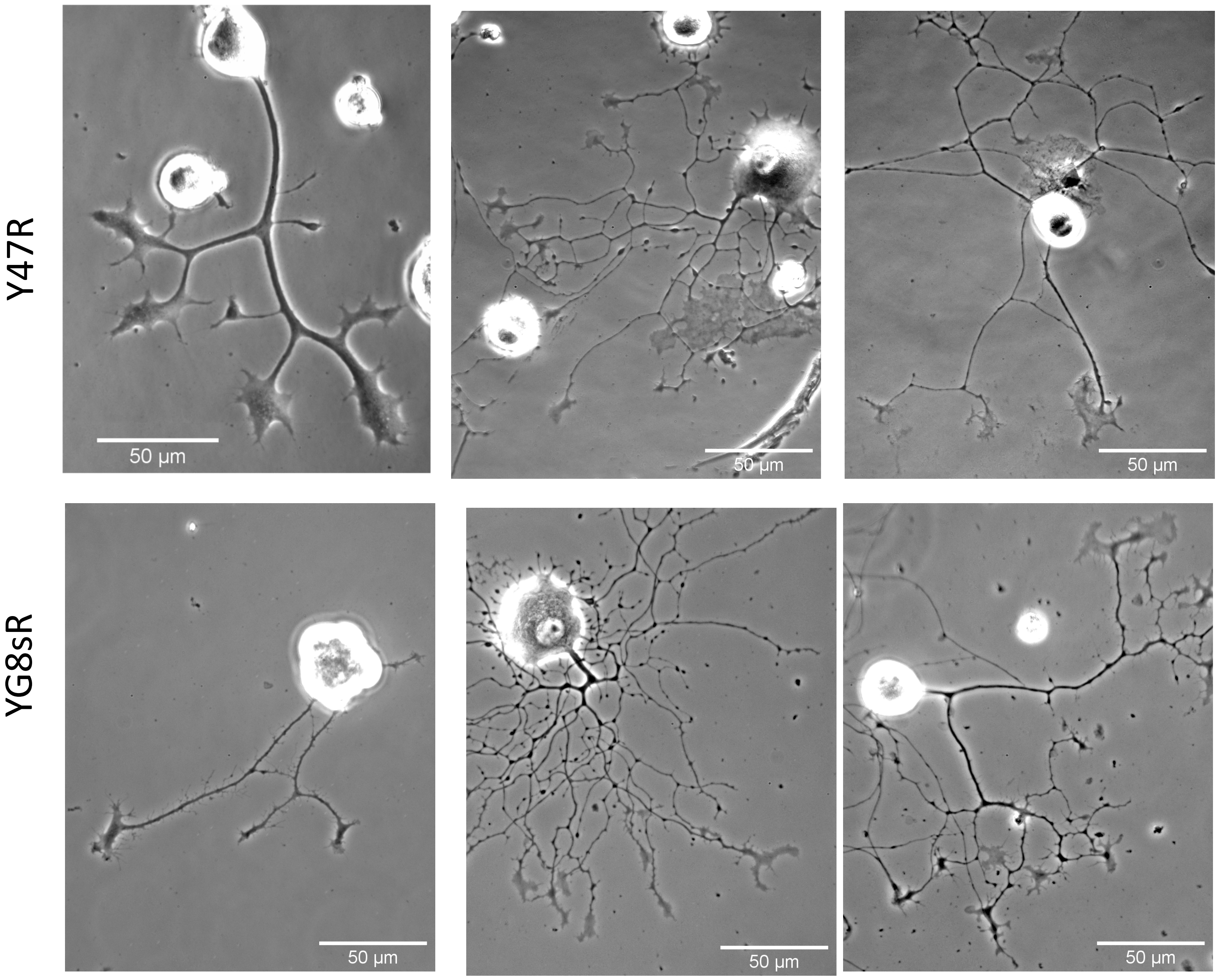

Figure S1: Images show representative sensory neurons growing in a typical primary culture of dorsal root ganglia from the control (Y47R) and YG8sR mice with 2 months of age. The heterogeneous morphology of sensory neurons and also their growth cones (at the most distal part of the neurites) is visualized here. Images were captured with Phase-contrast microscopy under in vivo conditions after 12-14 hours of culture using glass-bottom dishes. Scale bar 50 µm.
