# Supplementary material for: Frataxin Deficit Leads to Reduced Dynamics of Growth Cones in Dorsal Root Ganglia Neurons of Friedreich’s Ataxia YG8sR Model: A Multilinear Algebra Approach
Source: Front Mol Neurosci. 2022 Jun 13;15:912780. doi: 10.3389/fnmol.2022.912780 (PMC9236133; doi:10.3389/fnmol.2022.912780)
Supplement: Supplementary file 8 [file Image_2.pdf]

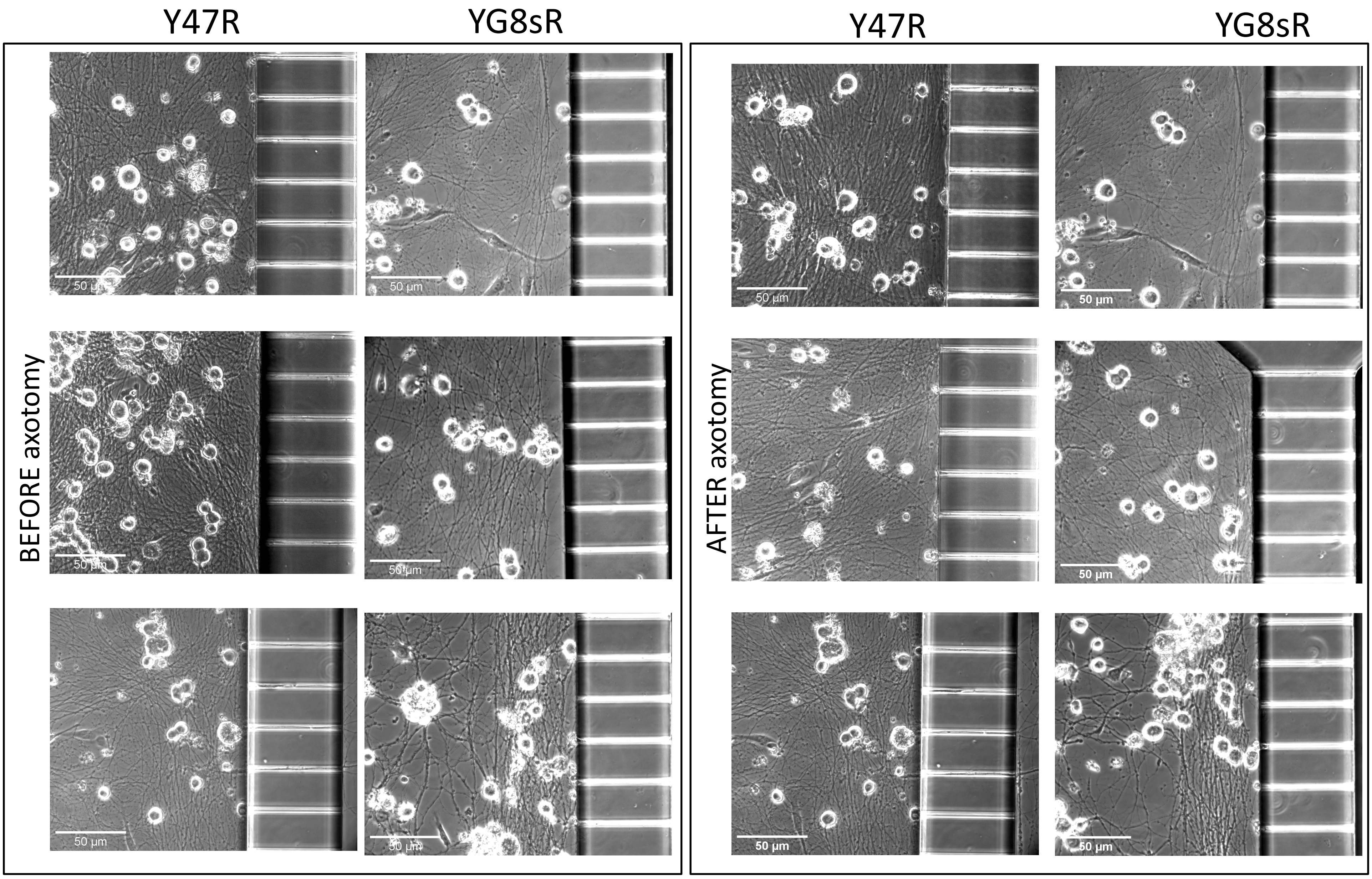

Figure S2: Images show sensory neurons of the control (Y47R) and YG8sR mice growing in the microfluidic chambers. Each image corresponds to an independent experiment. Images were captured with Phase-contrast microscopy under in vivo conditions. Images show how the neuronal somas are distributed in the SOMA chamber of the microfluidic device before (left panel) and after (right panel) the axotomy. Signs of cell death or loss of neuronal somas are not evident after the axotomy. Scale bar 50 µm.
