# Supplementary material for: Frataxin Deficit Leads to Reduced Dynamics of Growth Cones in Dorsal Root Ganglia Neurons of Friedreich’s Ataxia YG8sR Model: A Multilinear Algebra Approach
Source: Front Mol Neurosci. 2022 Jun 13;15:912780. doi: 10.3389/fnmol.2022.912780 (PMC9236133; doi:10.3389/fnmol.2022.912780)
Supplement: Supplementary file 9 [file Image_3.pdf]

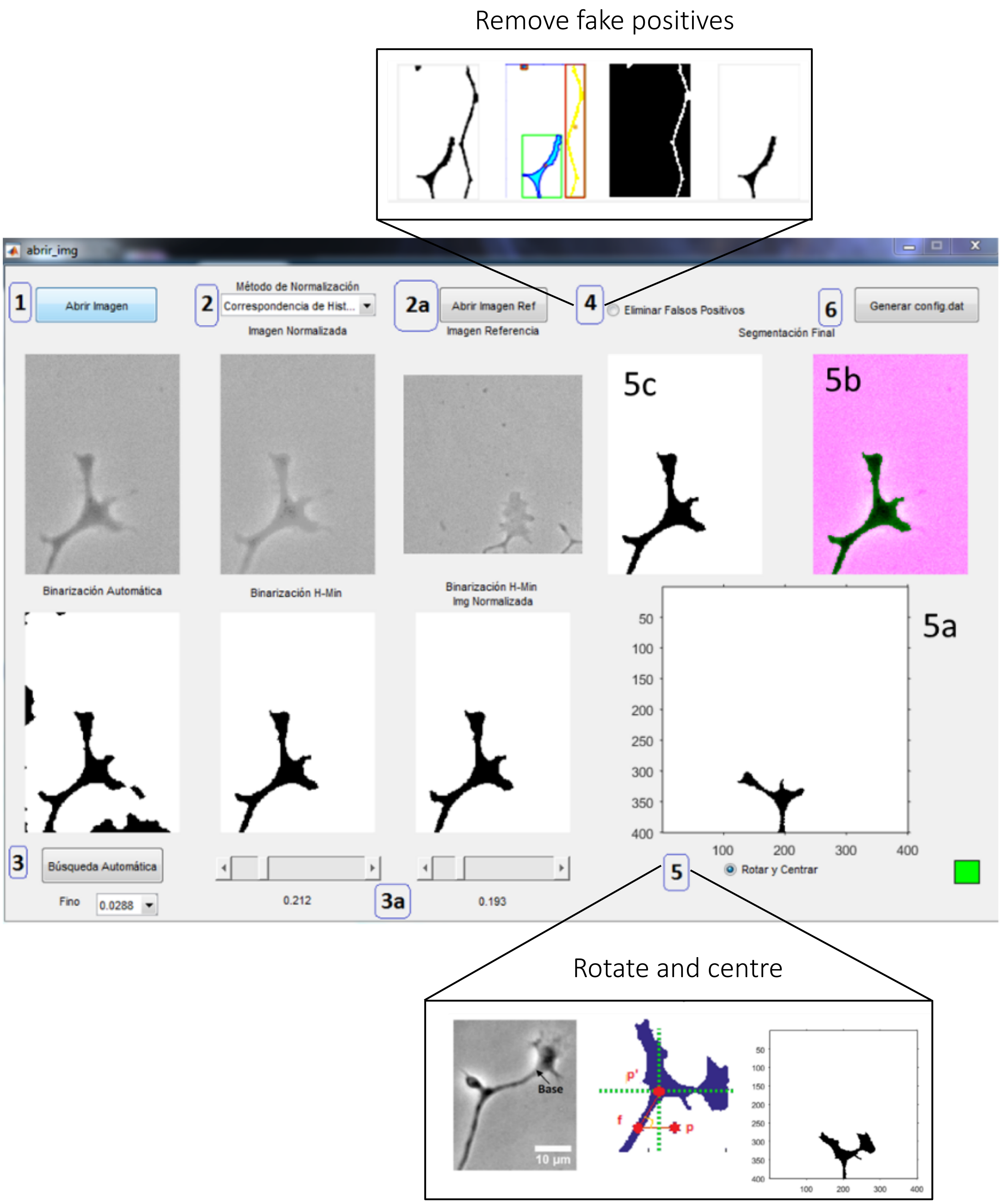

Figure S3: Platform to process phase-contrast time lapse experiments in MATLAB . Graphical user interface in MATLAB for obtaining semiautomated segmentation of time lapse experiments. For each time-lapse, the 6 following steps are followed. 1: Load an image. 2: Improve the discriminative information by applying a light normalization technique. 3: Search for the best threshold to binarise the image using the H-minima transform. 4: Elements not belonging to the region of interest are removed from the binarised image. 5: Rotate vertically and centre image horizontally to normalize the spatial position of the growth cone. 6: Parameter Processing: The parameters selected in the previous step and stored are used to segment a Time-Lapse, in this case, from 120 images.

The user interphase was created with Image Batch Processor. Image Batch Processor allows processing a group of images found in the same folder. Once a function is specified, the application processes all the images in the folder (time-lapse) or selected ones. For each image loaded, three binary images are obtained and compared with each other. Then, we obtain a defined binary image by looking for the proper black pixels present in at least two images and discarding the other.
