# Supplementary material for: Frataxin Deficit Leads to Reduced Dynamics of Growth Cones in Dorsal Root Ganglia Neurons of Friedreich’s Ataxia YG8sR Model: A Multilinear Algebra Approach
Source: Front Mol Neurosci. 2022 Jun 13;15:912780. doi: 10.3389/fnmol.2022.912780 (PMC9236133; doi:10.3389/fnmol.2022.912780)
Supplement: Supplementary file 10 [file Image_4.pdf]

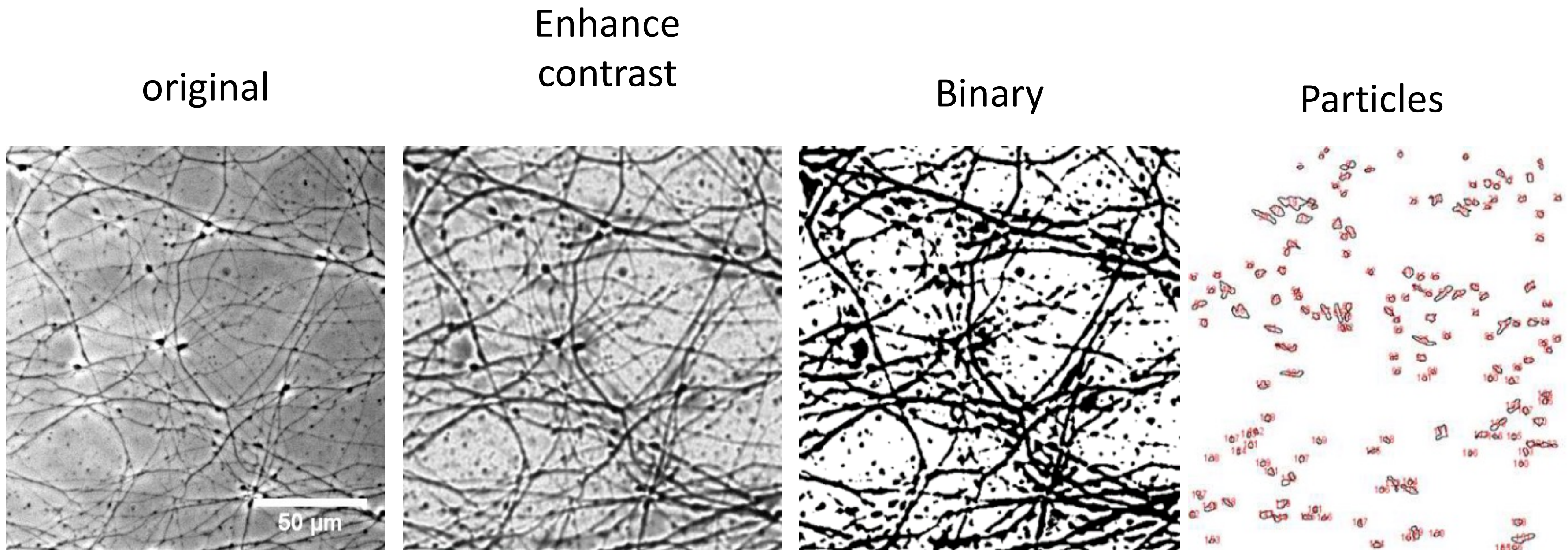

Figure S4: Pre-processing steps of phase contrast images to obtain fragmented and total area of the axons. Images show a visualization of the steps taken to enhance the contrast and resolution of phase-contrast images to obtain measurements of the area covered by axons. All the steps were performed with Fiji / ImageJ (N.I.H) software. A) The first step is to select a squared section (700X700 pixels) of the original image. B) Enhance contrast. C) Example of the binary image obtained. D) Black outlines and numbers(red) show the particles detected in the image. The total area of the axonal network corresponds to the total sum of pixels in (C), and the fragmented area corresponds to the sum of the area occupied by the particles in (D). Scale bar 50 µm.
