# Supplementary material for: Frataxin Deficit Leads to Reduced Dynamics of Growth Cones in Dorsal Root Ganglia Neurons of Friedreich’s Ataxia YG8sR Model: A Multilinear Algebra Approach
Source: Front Mol Neurosci. 2022 Jun 13;15:912780. doi: 10.3389/fnmol.2022.912780 (PMC9236133; doi:10.3389/fnmol.2022.912780)
Supplement: Supplementary file 11 [file Table_1.docx]

**Supplementary Table 1**: Time-Lapse overview experiments.

| **Age (months)** | **Genotype** | **Experiments analyzed** | **Total frames analyzed** |
| --- | --- | --- | --- |
|  |  |  |  |
| 2 | Y47R (control) | 40 | 4800 |
|  | YG8sR | 19 | 2280 |
| 6 | Y47R (control) | 28 | 3360 |
|  | YG8sR | 22 | 2640 |
